# Impact of low light intensity on biomass partitioning and genetic diversity in a chickpea mapping population

**DOI:** 10.3389/fpls.2024.1292753

**Published:** 2024-02-01

**Authors:** Muhammad Naveed, Urmil Bansal, Brent N. Kaiser

**Affiliations:** ^1^ Centre for Carbon, Water and Food, The University of Sydney, NSW, Australia; ^2^ School of Life and Environmental Sciences, The University of Sydney, NSW, Australia; ^3^ Sydney Institute of Agriculture, The University of Sydney, NSW, Australia; ^4^ Plant Breeding Institute, Cobbitty, The University of Sydney, NSW, Australia

**Keywords:** abiotic stress, biomass partitioning, chickpea, genetic diversity, low light, mapping population, phenological plasticity

## Abstract

With recent climatic changes, the reduced access to solar radiation has become an emerging threat to chickpeas’ drought tolerance capacity under rainfed conditions. This study was conducted to assess, and understand the effects of reduced light intensity and quality on plant morphology, root development, and identifying resistant sources from a Sonali/PBA Slasher mapping population. We evaluated 180 genotypes, including recombinant inbred lines (RILs), parents, and commercial checks, using a split-block design with natural and low light treatments. Low light conditions, created by covering one of the two benches inside two growth chambers with a mosquito net, reduced natural light availability by approximately 70%. Light measurements encompassed photosynthetic photon flux density, as well as red, and far-red light readings taken at various stages of the experiment. The data, collected from plumule emergence to anthesis initiation, encompassed various indices relevant to root, shoot, and carbon gain (biomass). Statistical analysis examined variance, treatment effects, heritability, correlations, and principal components (PCs). Results demonstrated significant reductions in root biomass, shoot biomass, root/shoot ratio, and plant total dry biomass under suboptimal light conditions by 52.8%, 28.2%, 36.3%, and 38.4%, respectively. Plants also exhibited delayed progress, taking 9.2% longer to produce their first floral buds, and 19.2% longer to commence anthesis, accompanied by a 33.4% increase in internodal lengths. A significant genotype-by-environment interaction highlighted differing genotypic responses, particularly in traits with high heritability (> 77.0%), such as days to anthesis, days to first floral bud, plant height, and nodes per plant. These traits showed significant associations with drought tolerance indicators, like root, shoot, and plant total dry biomass. Genetic diversity, as depicted in a genotype-by-trait biplot, revealed contributions to PC1 and PC2 coefficients, allowing discrimination of low-light-tolerant RILs, such as 1_52, 1_73, 1_64, 1_245, 1_103, 1_248, and 1_269, with valuable variations in traits of interest. These RILs could be used to breed desirable chickpea cultivars for sustainable production under water-limited conditions. This study concludes that low light stress disrupts the balance between root and shoot morphology, diverting photosynthates to vegetative structures at the expense of root development. Our findings contribute to a better understanding of biomass partitioning under limited-light conditions, and inform breeding strategies for improved drought tolerance in chickpeas.

## 1 Introduction

Chickpea (*Cicer arietinum* L.), a source of vegan protein, is extensively farmed between 20° to 40° latitudes in more than 50 countries, worldwide (Abbo et al., 2003; FAOSTAT, 2021). It is a long day plant, and grows well under certain light conditions, such as 16 h day length with red and blue light wavelengths, ranged 610-700 nm and 425-490 nm, respectively (Soltani et al., 2004; Pettai et al., 2005). However, a slight deviation in their levels may lead to modifications in central processes related to biochemistry, cell division, morphology, phenology, physiology, and so on (Fan et al., 2018; Yang et al., 2018). For example, reduction of photoperiod to 11-12 h delays flowering in chickpea by 120 to 150 days, whereas, variation in light quality and intensity, promotes competition for carbon gain among different plant parts (Woźny and Jerzy, 2007; Poudel et al., 2008; Yu et al., 2017).

Among abiotic stresses, light or solar radiation is the leading factor that regulates plant growth and expansion under any environment (Shafiq et al., 2021). Three components of light i.e. quality, intensity, and photoperiod, largely determine a plants’ photosynthetic capacity, establishment, and yielding ability (Khalid et al., 2019). Chickpea, being a rainfed crop, has major cultivation under subtropical and Mediterranean zones (Chen et al., 2017). Drought and heat are the characteristic features of these climates, and have long been considered as major yield constraining factors. Suboptimal light intensity and quality, caused by various climatic events (prolonged cloud cover, foggy weather etc.), and cultural practices, is now becoming an emerging challenge to sustainable chickpea production under these environments (Jha et al., 2014; Naveed, 2022). Because, this triggers unbalanced partitioning between root and shoot morphology due to variation in interception of photosynthetic photon flux density (PPFD), and underlying processes regulating plant growth and expansion (Park and Runkle, 2017). As in the study by Gao et al. (2017) on maize, they observed that plants reallocate a greater proportion of photosynthetic resources to above-ground organs, reducing the root-to-shoot ratio. This shift led to abnormal plant structure and increased lodging. More precisely, the elongation of petioles and stems leads to a decrease in leaf size and thickness, along with an increase in internode length, ultimately resulting in reduced stem thickness, weakened structural integrity, and diminished shoot biomass (Okoli and Wilson, 1986; Su et al., 2014; Liu et al., 2018). Likewise, low light effects on root biomass are also extreme. Lake and Sadras (2014) in an experiment on chickpea, and Sparkes et al. (2008) on wheat, reported more than two fold decrease in root-length density, diameter, absorption area, and root biomass under low light compared to control treatment. This reduced root growth could constrain a plants ability to extract water deeper from the soil layers (Kashiwagi et al., 2015; Gao et al., 2017). We lack information on these aspects in chickpeas, as the available literature predominantly covers soybean and other crops. Total plant biomass, particularly the biomass of roots and shoots, is a crucial adaptive strategy in water-limited conditions. This may be a potential factor contributing to low productivity under suboptimal light in rainfed agricultural systems (Green-Tracewicz et al., 2011; Liu et al., 2018).

Breeding for low light tolerance is the most effective strategy for mitigating these yield losses (Rai et al., 2021). This approach can lead to the development of light-insensitive genotypes capable of maintaining their natural traits even in challenging environments through enhanced light interception, and photosynthetic ability. Mapping populations provide a valuable toolbox that integrates genomics with breeding, and related disciplines to identify desirable recombinants (Aryamanesh et al., 2010; Scott et al., 2020). To exploit all these, we need comprehensive knowledge of chickpea plant responses, traits variability, and underlying genetic mechanisms controlling targeted indices under these environments (Tardieu and Tuberosa, 2010; Choudhary et al., 2018; Ao et al., 2019). Contrastingly, in prior field trials, we observed variations in chickpea yield and the responses of various growth parameters across years, driven by distinct climatic conditions and varying levels of solar radiation (Kaloki, 2017). Higher yields of 46% to 54% were recorded during mostly sunny growing seasons, while overcast conditions resulted in taller plants (7 cm to 10 cm) with an overall lower yield (Naveed, 2022). This behavior could be due to the onset of the shade avoidance mechanism, which has more significant effects on crop root architecture and biomass accumulation of cultivars (Franklin, 2008; Green-Tracewicz et al., 2011). These adjustments might compromise chickpeas’ ability to tolerate water-deficit conditions. This study aimed to achieve the stated goals using a Sonali/PBA Slasher mapping population under controlled environmental conditions.

## 2 Materials and methods

### 2.1 Plant material and experimental design

This study was conducted in 2020 at the Plant Breeding Institute of the University of Sydney, Narrabri campus, NSW, Australia. The plant material comprised 180 test entries and included 176 RILs, which were developed using two drought tolerant commercial lines, Sonali as a female while PBA Slasher as a male parent (Kaloki, 2017). In addition, two high yielding and disease resistant commercial cultivars, PBA Seamer and PBA Striker, were used as standard checks (Vance et al., 2021). This mapping population has a range of variation for some of the plant traits such as architecture, cropping period, and plant biomass useful for conferring drought tolerance in chickpea (Ramamoorthy et al., 2016; Maqbool et al., 2017; Ramamoorthy et al., 2017). Details of entries along with characteristic features are given in **Supplementary Table 1**. All these lines were evaluated under two light treatments in a fully replicated trial, being laid out inside two growth chambers of a glasshouse. The experimental pots were placed side-by-side on two parallel benches, facing north and south, and separated by an entryway. Northern bench comprised natural light (NL), while southern consisted of low light (LL) treatment. The design used was split-block with four replications, and included 1440 pots. These were randomized in twenty rows by 36 columns, with each replicate block of 180 genotypes comprising ten rows by 18 columns, using DiGGer package of R software (Coombes, 2009). Two replicates were placed in each growth chamber under each treatment, with every pot assigned a unique identification number. This process was completed with utmost care to avoid any type of error.

For potting purpose, soil (rich in clay) and sand were mixed together in a 3:1 ratio, respectively, to fill the pots with 9×9×20 cm diameter. All the pots were watered until dripping to ensure the soil had enough moisture contents on the day of sowing. Two seeds, 3 cm deep and 5 cm apart, were sown per pot. Seeds, which germinated and cracked through the soil surface first, were retained in each pot while others, once emerged, were pulled out with caution immediately. All the plants in both treatments were fertilized (N 6.1%, P 12%, K 22.5%, S 2.2%, Zn 0.55%) with Cotton Sustain (Incitec Pivot Fertilisers, Australia) 10 days after sowing at a rate of 0.3 g per pot. On the same day, the plants were inoculated with a peat-based inoculant of Rhizobia (Nodule N, New Edge Microbials, Albury, Australia) to establish root symbiosis, and promote root nodulation. This was achieved by diluting 20 g of inoculum in 5 L of water, and distributing it to all 1440 pots uniformly. Further, a dose of liquid fertilizer (Thrive, Yates Australia, Padstow, Australia) enriched with NPK (25:5:8.8) and micronutrients (S 4.6, Mg 0.5, Fe 0.18, B 0.005, Cu 0.005, Zn 0.004, Mo 0.001) was applied 24 days after sowing to overcome any micronutrient deficiencies. All the plants were watered regularly to avoid water stress, and were staked upright when the shoot started bending or falling. Reverse cycle air conditioning was used to maintain daily day/night temperatures of both the growth chambers at 24 ± 2/16 ± 2°C, respectively. Whereas dehumidifiers (Quest series) were used to control humidity, which was set 50% to 70% to avoid mold or any other fungal disease incidence.

### 2.2 Treatments

Two light treatments i.e. natural light (NL) and reduced/low light (LL), were used to raise plants from seed sowing up to anthesis stage. Plants were harvested once anthesis commenced. The LL treatment was created using a mosquito net (1.2 mm mesh), covering the top and sides of one bench in each growth chamber. Seeds in both the treatments and growth chambers were sown once the benches allocated for LL treatment were covered with mosquito nets. Readings on light parameters were made starting at 11 am, which indicated a reduction of ~70% light in LL compared to NL treatment (**Supplementary Table 2**).

### 2.3 Measurement of available light

Readings on photosynthetic photon flux density (PPFD), red (R), and far-red (FR) light received by plants in both glasshouse chambers were done at five different experimental stages, and commenced at 11 am. At each stage, six measurements were performed on the same genotypes, comprising both parents, Sonali and PBA Slasher, two commercial cultivars, PBA Seamer and PBA Striker, and two RILs, 1_17 and 1_50, representing different positions in each replication of a treatment. First measurement was done a day before seed sowing, whereas the second was taken a week after seed sowing, followed by at 3, 4-leaf, and anthesis stages. PPFD measurements were carried out using AP4 Porometer (Delta-T Devices Ltd, Cambridge, UK) by holding the light sensor about 10 cm above the canopy. Whereas R and FR light measurements were performed using LightScout red/far red meter (Spectrum Technologies, Inc., Aurora, IL, USA). Red and far-red light measurements were assessed right after estimating PPFD by placing the sensor at the same spot as the PPFD sensor, and writing down the values, immediately.

### 2.4 Measurement of photosynthetic rate of parents

Because of the small window, it was not feasible to measure the photosynthesis of 180 genotypes in four replicates and two treatments. Therefore, it was assessed only for parents, Sonali and PBA Slasher. Measurements were done at three-growth stages viz., 3-leaf stage, 4-leaf stage, and at anthesis using a portable CIRAS-3 machine (PP Systems, Amesbury, MA, USA). This photosynthesis system has a leaf cuvette of 4.5 cm^2^, and can set light closest to the approximation of sunlight (38% red, 37% green, and 25% blue) using light-emitting diodes. The flow rate set was 400 cc min^-1^, and the reference CO_2_ at 400 μmol mol^-1^. These measurements were performed between 9:00 am and 4:00 pm by selecting fully developed youngest leaves. At each selected stage, all photosynthetic measurements were done four times apiece on both the parents at growing PPFD, starting from zero to 1500 μmol m^-2^ s^-1^. PPFD increased by 100-μmol m^-2^ s^-1^ every time for 16 levels. The actual values of photosynthetic rate were adjusted to the real chickpea leaf area, which was estimated using ImageJ software.

### 2.5 Phenotyping

Following traits were measured in eight (4 + 4) replicates under NL and LL treatments as per procedure explained in **Supplementary Table 2**, and used by previous researchers (Ali et al., 2010; Walia et al., 2020; Naveed, 2022).

#### 2.5.1 Days to emergence (DTE)

#### 2.5.2 Days to first floral bud (DFFB)

#### 2.5.3 Days to anthesis (DTA)

#### 2.5.4 Plant height (PH)

#### 2.5.5 Nodes per plant (NPP)

#### 2.5.6 Internodal length (IL)

#### 2.5.7 Branches per plant (BPP)

#### 2.5.8 Shoot dry biomass per plant (SDBPP)

#### 2.5.9 Root dry biomass per plant (RDBPP)

#### 2.5.10 Root to shoot ratio (RSR)

#### 2.5.11 Plant total dry biomass (PTDB)

### 2.6 Statistical analysis

All statistical analyses such as ANOVA, correlation, and principal component reported herein were performed on traits given in section 2.5 using “Genstat” computer software version 16.0 (Payne et al., 2011). Treatment effects (individual and interactive) were estimated (*P* < 0.05) using function “RML linear mixed model” and comprised genotypes (G), and treatments (T) as fixed-terms, whereas replications within treatments, as random-terms. For the measurements done over time, such as photosynthesis, genotypes (G), treatments (T), and growth stages (GS) were used as fixed-terms, and replications within treatments as random-terms. We preferred Wald test (also known as Wald Chi-Squared test) over others because it is based on parametric statistical measures, and provide information, collectively, on the significance of a set of independent variables in a model. It is simple, quicker, and can add or remove the parameters for certain explanatory variables depending upon their contribution in the model (Arango-Botero et al., 2023). Heritability in broad-sense (H^2^_B.S._) was also worked out for all the traits using formula given by Nyquist and Baker (1991). H^2^ was considered as high (> 60%), moderate (30-60%), and low (< 30%), as per Johnson et al. (1955). Correlation coefficients among various indices such as DTE, DTFFB, DTA, PH, IL, NPP, BPP, SDBPP, RDBPP, RSR, and PTDB, in NL and LL conditions were computed following Pearson’s technique. Principal component analysis was also done on the same parameters using the multivariate analysis function of Genstat software. Principal components with > 1 eigenvalues were tabulated, and used to construct biplot using the same software. The curves were fitted on photosynthetic rate over time against growing PPFD (detail given in section 2.4) captured at selected three growth stages under NL and LL treatments, using nonlinear polynomial regression function of software GraphPad prism version 7.0 (GraphPad Software incorporation, USA) (Schneider et al., 2012).

## 3 Results

In the present study, changes in optimum light intensity, critically impacted rate of photosynthesis, and carbon gain of genotypes. This created a competition among different plant organs to intercept maximum light, resulting in, modified shoot and root structures. Specifically, plant biomass was reduced, and this reduction was greater for roots compared to shoots. In addition, shoots become thinner, longer, and prone to breakage. Still, few entries in this mapping population performed better, and exhibited phenotypic plasticity across environments. Greater heritability values for some of the desired traits, PTDB, RDBPP, and SDBPP suggested their potential utilization, and scope of improvement in breeding programs. Detailed results are presented in the following subsections.

### 3.1 Analysis of variance for recorded traits

The variance analysis revealed significant differences between both light treatments. Low light resulted in 72% reduction in PPFD, implying that only 28% ambient light was available to plants to carry various gas exchange processes. Similarly, the optimum light quality for normal plant growth in the form of red, far-red, and their ratios was deteriorated by 71%, 71% and 2%, respectively (**Supplementary Table 3**). Two factor ANOVA for G, T and their interaction showed significant (*P* < 0.001) differences for traits like DTE, DTFFB, DTA, PH, IL, NPP, BPP, SDBPP, RDBPP, RSR and PTDB except BPP which was non-significant even at *P* > 0.05 (**Supplementary Table 5**). Higher estimates of G than G×E suggested larger genotype effects than environments on the traits investigated.

### 3.2 Impact of light treatments on parental photosynthesis

**Figure 1:**
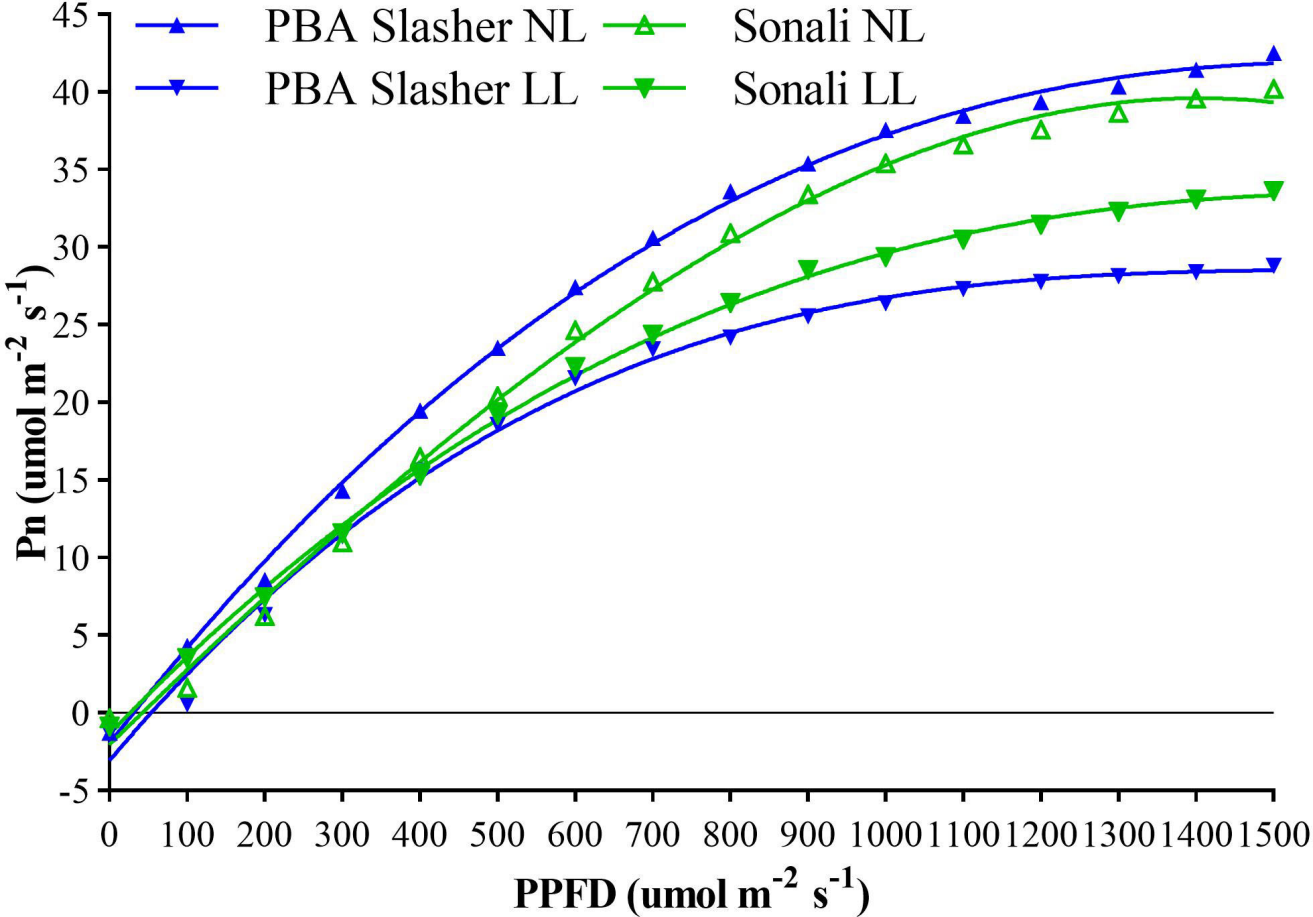
Association between photosynthesis (Pn) and photosynthetic photon flux density (PPFD) of Sonali and PBA Slasher recorded over different growth stages under natural light (NL) and low light (LL) treatments.

Effects of light treatments (T) on net photosynthetic rate of parental genotypes (G), assessed at different growth stages (GS), revealed significant differences between T (*P* < 0.001), and G×T interaction (*P* < 0.05), as indicated in **Supplementary Table 4**. Effects of LL on Pn or carbon gain were greater for PBA Slasher, and reduced its net photosynthetic rate by 38.8% compared to 14.8% of Sonali. Pn for PBA Slasher was greater under NL, and for Sonali under LL treatments (**Supplementary Table 4**). The fitting of polynomial regression curves revealed 53.5% variation for this trait. Initially, Pn was negative at zero PPFD, while it increased gradually from growing PPFD of 100 umol m^-2^ s^-1^, and reached highest at 1500 umol m^-2^ s^-1^ under NL and LL treatments (**Figure 1**).

### 3.3 Response of RILs and parents to varied light conditions

Alteration in quality and quantity of light had altered the expression of majority of the indices under both light treatments (**Table 1**). Comparison of trait means of Sonali in NL vs LL revealed a reduction in DTE, DTFFB, SDBPP, RDBPP, RSR and PTDB, and an increase in DTA, PH, IL and NPP, with no effect recorded on BPP under LL. For PBA Slasher, estimates of DTFFB, NPP, BPP, SDBPP, RDBPP, and RSR were reduced, whereas, for DTE, DTA, PH, and IL increased in LL environment. Overall, trait means for DTE, SDBPP, RDBPP, RSR, and PTDB were greater under NL than DTFFB, DTA, PH, IL, NPP, and BPP, which were higher under LL treatment. The range of variation as indicated by CV% was 54.6% to 8.3% in LL, and 56.1% to 6.3% under NL conditions. It was highest for RDBPP (54.6% vs 37.5%), PTDB (32.7% vs 28.9%), and BPP (50.7% vs 56.1%) in LL vs NL environments, respectively, which showed their potential use, and possibility of further improvement.

**Table 1:** Various statistical measures of genotypes (parents & RILs) on plant traits recorded at anthesis stage, and impact of natural light (NL) and low light (LL) treatments on their expression (% increase/decrease) inside a glasshouse.

Traits	NL					LL					% change	H^2^ B.S. %
	Sonali	Slasher	Mean ± SEM	RILs range	CV%	Sonali	Slasher	Mean ± SEM	RILs range	CV%		
DTE	15.3	6.8	6.45 ± 0.13	4.5-15	27.5	8.0	8.0	6.21 ± 0.11	4.3-15.5	23.0	-3.7	61.4
DTFFB	39.5	34.3	33.2 ± 0.47	21.5-56.5	19.1	39.0	32.5	36.3 ± 0.58	21.8-70.8	21.5	9.2	80.6
DTA	48.8	55.0	43.8 ± 0.71	29.5-68	21.9	56.8	56.3	52.2 ± 0.86	29.8-85	22.0	19.2	81.2
PH	39.5	33.0	37.5 ± 0.44	25.3-56.5	15.8	50.5	48.6	52.4 ± 0.61	28.5-74.6	15.5	39.9	77.8
IL	2.1	1.5	1.82 ± 0.01	1.53-2.77	6.3	2.5	2.2	2.43 ± 0.02	2.24-4.8	8.3	33.4	36.1
NPP	20.5	22.8	20.6 ± 0.22	10.5-27.8	14.3	21.0	21.8	21.6 ± 0.22	8.5-27.3	13.7	4.9	77.2
BPP	4.3	10.0	6.12 ± 0.26	1.0-21.3	56.1	4.3	9.3	6.22 ± 0.24	1.0-16.8	50.7	1.7	72.4
SDBPP	1.1	1.2	1.09 ± 0.02	0.62-2.3	23.3	0.8	0.6	0.78 ± 0.01	0.22-1.44	23.2	-28.2	75.6
RDBPP	0.8	1.2	0.78 ± 0.02	0.24-2.01	37.5	0.4	0.2	0.37 ± 0.02	0.1-1.01	54.6	-52.8	75.1
RSR	68.3	97.5	69.7 ± 0.97	31.4-96.7	18.6	53.5	35.8	44.4 ± 1.08	19.3-88.9	32.7	-36.3	67.9
PTDB	1.9	2.4	1.87 ± 0.04	0.97-4.09	28.9	1.2	0.7	1.15 ± 0.03	0.34-2.38	32.7	-38.4	76.8

DTE, Days to emergence (days); DTFFB, Days to first floral bud (days); DTA, Days to anthesis (days); PH, Plant height (cm); IL, Internodal length (cm); NPP, Nodes per plant; BPP, Branches per plant; SDBPP, Shoot dry biomass per plant (g); RDBPP, Root dry biomass per plant (g); RSR, Root/shoot ratio (%); PTDB, Plant total dry biomass (g).

### 3.4 Impact of low light on plant traits captured

It was assessed through % increase/decrease using trait means under NL and LL treatments (**Table 1**). Overall, chickpea seedlings took fewer days to emerge (-3.7%) under LL, but showed greater reduction in RDBPP (-52.8%), PTDB (-38.4%), RSR (-36.3%), and SDBPP (-28.2%) once harvested at anthesis stage. However, these took more days to develop first floral buds (9.2%), and to commence anthesis (19.2%), but with greater PH (39.9%), IL (33.4%), NPP (4.9%), and BPP (1.7%) compared to NL treatment.

### 3.5 Appraisal of heritability (H^2^) values for traits investigated

The use of desirable plant traits in any breeding scheme depends upon their heritability values. The estimates of broad-sense heritability in this study were moderate to high, and ranged from 36.1% to 81.2% (**Table 1**). For all the traits (DTA, DTFFB, PH, NPP, TBP, SBPP, RBPP, BPP, RSR, and DTE) except IL, these were greater than 50% suggesting that variability in respective traits were due to genetic differences among plant material. For IL, it was lowest with 36.1%, implying greater environmental influence.

### 3.6 Association among different plant traits

All the parameters recorded under NL and LL treatments revealed positive and significant correlation coefficients with few exceptions (**Supplementary Table 6**). In NL, DTE was positively and significantly associated with DTFFB (r= 0.63) and DTA (r= 0.76). Likewise, the association of DTA with PTDB (r= 0.88), RSR (r= 0.81), RDBPP (r= 0.90), SDBPP (r= 0.84), BPP (r= 0.60), NPP (r= 0.93), IL (r= 0.17), and PH (r= 0.94) was positive and significant. The association of PTDB with RDBPP (r= 0.99) and SDBPP (r= 0.99) was near to one because it was estimated by adding both shoot and root biomass. The relationship of IL with DTFFB and RDBPP was positive, whereas with NPP and RSR, it was negative, but non-significant. Except these, the other correlation coefficients determined among other traits like DTE, DTFFB, DTA, PH, IL, NPP, BPP, SDBPP, RDBPP, RSR and PTDB were positive and significant. Under LL treatment, except for the association of IL with NPP, which was negative and non-significant, all other correlation coefficients among different parameters were positive, significant, and similar to those obtained under NL conditions, therefore, not repeated here. However, values of coefficients for most of the traits in LL treatment were higher than the corresponding ones obtained under NL conditions.

### 3.7 Principal component analysis (PCA) for plant traits and genotypes

Prior to running PCA, we explored our data through descriptive statistics, and correlation analysis to cognize its characteristics, and address PCA limitations. Firstly, we identified outliers in the residual table, removed them using the masking tool, and run the recalculation to automatically update the output. Secondly, PCA presume that the data is linear, and in case of non-linear, will not detect underlying structure properly. Therefore to ensure equal weight and influence of each of the variable, we standardized it using Z-scores. Thirdly, sample size is very important for reliable, and robust PCA analysis. Mostly, a small input file can lead to misleading pattern/correlation between variables due to sampling error (Shaukat et al., 2016). Whereas, this probability will vanish with increasing sample size (Björklund, 2019). Generally, it is recommended that data set should have at least 150 samples (Sofroniou and Hutcheson, 1999) or larger than five times the number of variables for valid results (Hatcher, 1994). Our data set comprising 1440 cases, completely fulfilled this requirement. Fourthly, to validate results, reliability and robustness is crucial in PCA, and to check it, we permuted one variable at a time, and kept the others as fixed i.e. independently, and sequentially (Linting et al., 2011; Storm, 2012).

**Figure 2:**
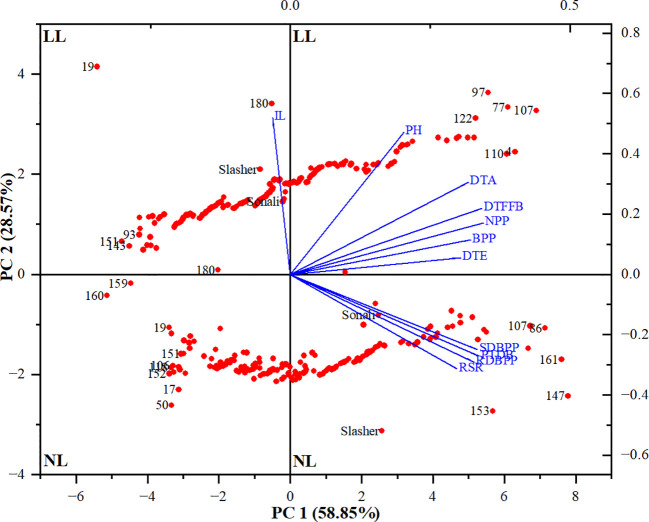
Biplot between principal components 1 and 2 (PC1 & PC2) showing the contribution of different traits (blue font) and genotypes (red font) in total variability under natural light (NL) and low light (LL) treatments. DTE, Days to emergence (days); DTFFB, Days to first floral bud (days); DTA, Days to anthesis (days); PH, Plant height (cm); IL, Internodal length (cm); NPP, Nodes per plant; BPP, Branches per plant; SDBPP, Shoot dry biomass per plant (g); RDBPP, Root dry biomass per plant (g); RSR, Root/shoot ratio (%); PTDB, Plant total dry biomass (g).

Afterwards, the PCA performed across both light treatments, collectively, explained 87.4% (PC1 = 58.9% & PC2 = 28.6%) of the total variation observed in Sonali/PBA Slasher RILs population (**Supplementary Table 7**). Except IL, all other parameters (NPP, DTFFB, PTDB, SDBPP, RDBPP, BPP, DTA, DTE, RSR, and PH) shared positive scores on PC1, ranging from 35% to 20%, respectively. In contrast, PC2 was largely influenced by IL (52%), and PH (47.3%) with positive, and RSR, RDBPP, PTDB and SDBPP, with negative values (-31.4% to -24.9%). A genotype-by-trait biplot constructed between PC1 and PC2 displayed indices with positive associations (< 90°), independent or no associations (= 90°), and negative associations (> 90°) based on the angle between them (**Figure 2**). It identified positive correlations among vegetative (BPP, NPP, and PH), phenology (DTE, DTFFB, and DTA), and biomass (SDBPP, PTDB, RDBPP, and RSR) parameters. However, relationship of IL with biomass indices, and that of PH with RSR was found to be negative. Overall, association among biomass parameters seemed stronger than vegetative, and phenological traits. Nevertheless, positively correlated all the traits contributed more towards the LL tolerance of genotypes, so can be selected as markers at anthesis stage in chickpea.

**Figure 3:**
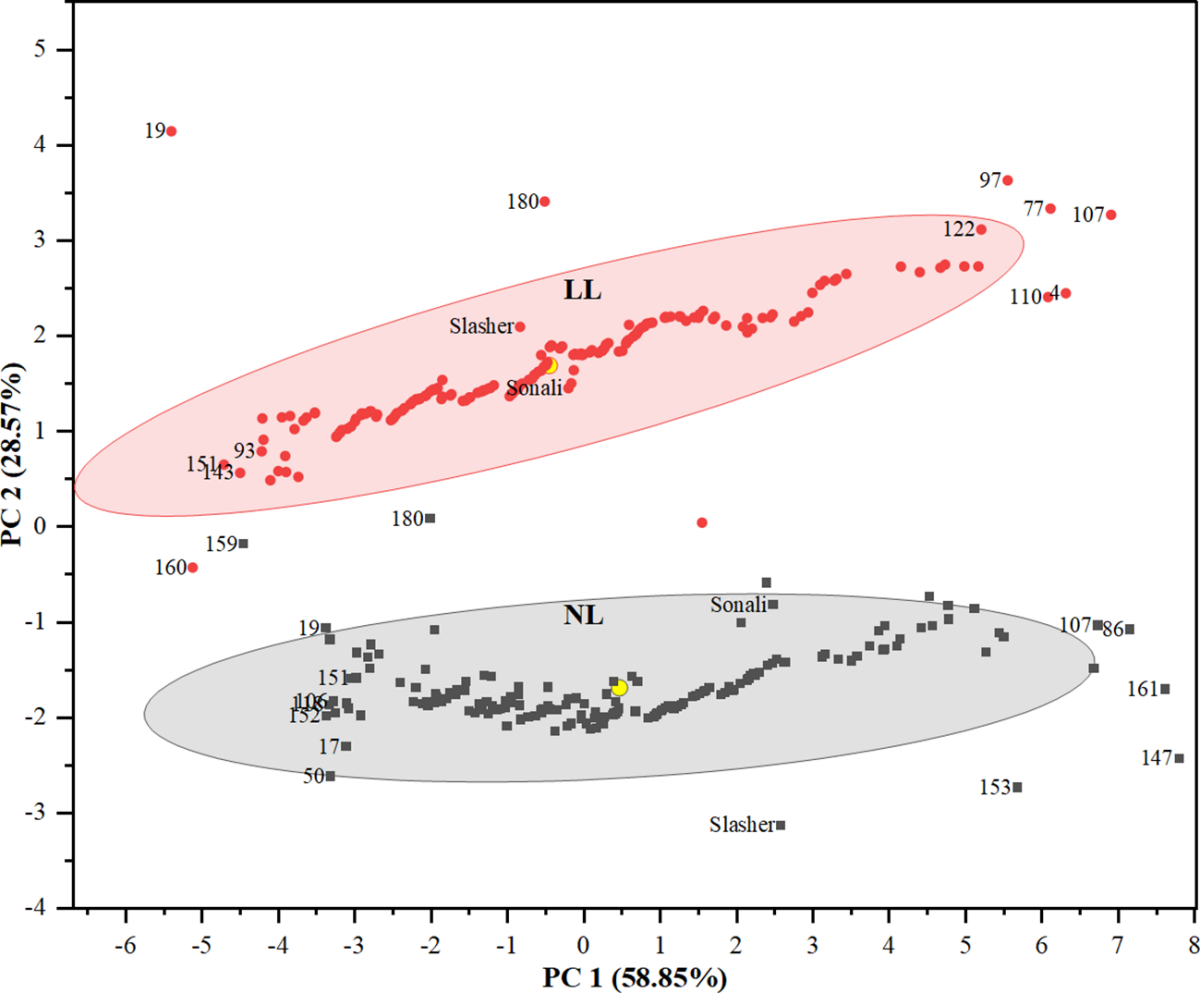
Clustering of genotypes in a PCA biplot (PC1 & PC2) showing the position of different genotypes from centroid (yellow circle) under natural light (NL) and low light (LL) treatments.

To assess the performance of genotypes, PCA biplot identified entries, #147, #161, #153, #86, and #107 as distant with strong positive association with carbon gain indicators i.e. SDBPP, PTDB, RDBPP, and RSR (**Figures 2**, **3**), on NL quadrant. These entries contributed the highest values for these traits on PC1. Entries, #50, and #17 were recognized as far-off but with negative associations, and minimum scores for biomass parameters. The rest of the genotypes might have low to medium values for these indices. All the vegetative, and phenological traits occupied LL quadrant, where entries #107, #77, #97, #122, #4, and #110 suggested strong positive association, and maximum values for these traits on PC2. This biplot also displayed entries #19, #180, and #151 as away from origin with strong negative correlation, and lowest share for biomass indices under LL. This is also evident from clustering of entries between both light treatments, and distances from centroid in each environment, indicating their share in phenotypic variance (**Figure 3**). Between parents, PBA Slasher influenced both the environments with greater percentage than Sonali. This share was much higher in NL compared to LL treatment.

### 3.8 Best versus poor performing genotypes identified in PCA biplot

The superior and underperforming entries, given in section 3.7, were further assessed for biomass and anthesis period, being vital for drought tolerance in chickpea (**Table 2**). Among those, entries #147, #161, #153, and #107 initiated anthesis, on average, in about 56 to 66 days (DTA) in NL, and accumulated highest PTDB (4.09 to 3.12 g), RDBPP (2.01 to 1.38 g), SDBPP (2.08 to 1.74 g) with greater RSR (96.5 to 79.3%), respectively. In comparison, light-sensitive entries viz., #118, #106, #151, and #159 were early into flowering (~30 to 31 days), and produced less than half PTDB, RDBPP, SDBPP with lowest RSR than best entries under the same environment. Under LL, top entries, #107, #4, #110, and #122, in pursuit to adapt to prevailing conditions, delayed anthesis, on average, by ~10 days compared to best performing genotypes under NL, and by more than double to underperforming entries, #93, #143, #151, and #160, in reduced light treatment. Therefore, values of PTDB (2.38 to 2.01 g vs 0.55 to 0.34 g), RDBPP (1.01 to 0.79 g vs 0.14 to 0.12 g), SDBPP (1.44 to 1.22 vs 0.41 to 0.22 g), and RSR (79.3 to 65.1% vs 56.5 to 34.0%) were bigger for best genotypes than others in this environment, respectively. Estimates of parents for most of these traits were between best and poor performing RILs, implying presence of desirable recombinants.

**Table 2:** Trait means of selected four superior and four poor performing recombinant inbred lines based on accumulated plant total dry biomass (PTDB) across two light treatments.

Entry	Natural light (NL)					Entry	Low light (LL)				
	PTDB	RDBPP	SDBPP	RSR	DTA		PTDB	RDBPP	SDBPP	RSR	DTA
147	4.09	2.01	2.08	96.7	56.3	107	2.38	0.94	1.44	65.6	75.0
161	3.83	1.71	2.12	80.5	56.3	4	2.36	1.01	1.36	74.3	75.0
153	3.82	1.52	2.30	65.8	56.3	110	2.27	1.01	1.27	79.3	74.8
107	3.12	1.38	1.74	79.3	66.3	122	2.01	0.79	1.22	65.1	74.8
*Sonali*	*1.86*	*0.75*	*1.11*	*68.0*	*48.8*	*Sonali*	*1.23*	*0.42*	*0.81*	*52.0*	*56.8*
*Slasher*	*2.42*	*1.20*	*1.23*	*97.5*	*55.0*	*Slasher*	*0.74*	*0.20*	*0.55*	*35.8*	*56.3*
118	1.05	0.29	0.76	37.9	30.5	93	0.55	0.14	0.41	34.6	34.3
106	1.04	0.38	0.66	56.8	32.3	143	0.52	0.13	0.39	34.0	33.8
151	1.02	0.37	0.65	56.5	32.0	151	0.50	0.14	0.36	39.1	33.3
159	1.00	0.24	0.76	31.9	31.3	160	0.34	0.12	0.22	56.5	29.8
*Mean*	*2.33*	*0.99*	*1.34*	*67.1*	*46.5*	*Mean*	*1.29*	*0.49*	*0.80*	*53.8*	*54.4*
*SEM*	*0.41*	*0.21*	*0.21*	*7.02*	*4.29*	*SEM*	*0.27*	*0.13*	*0.15*	*5.42*	*6.3*
*SD*	*1.30*	*0.66*	*0.66*	*22.2*	*13.6*	*SD*	*0.87*	*0.40*	*0.48*	*17.1*	*19.9*

PTDB, Plant total dry biomass (g); RDBPP, Root dry biomass per plant (g); Shoot dry biomass per plant (g); RSR, Root/shoot ratio (%); Days to anthesis (days); SEM, Standard error of mean; SD, Standard deviation from mean.

Entries given above parents (Sonali & Slasher) produced highest PTDB, whereas below lowest.

## 4 Discussion

The uncertain chickpea production under variable environmental conditions is often attributed to multiple biotic and abiotic stresses (Shah et al., 2020). Breeding for improved cultivars against these factors requires investigation of all possible causes responsible for low and unstable chickpea yields (Maqbool et al., 2017). Effects of shade or LL on different agro-morphological plant traits are least investigated in chickpea. However, to formulate any breeding strategy, assessment of plant responses, and trait variability are one of the preliminary steps, and the major objectives of this study (Chen et al., 2017). It was revealed on reviewing literature that no such study was conducted in chickpea, previously, that specifically investigated effects of LL on morphology, phenology, and physiology at the anthesis stage. Apparently except light availability, this experiment was conducted under favorable temperature, moisture, and nutrients supply, hence, potential effects determined were mostly by light composition.

### 4.1 Evaluation of methodology executed for inducing low light environment

Differences observed in PPFD, RL, FRL, and RL/FRL ratio between two light treatments were significant, suggesting that the method used to mimic reduced light conditions in this study had successfully simulated the LL environment. Previous studies had also reported significant differences in quality of light, induced using different procedures. Li et al. (2010) in a field study on chickpea used a black commercial shade cloth to create LL environment up to vegetative phase, and found 45% decrease in incident PPFD. In soybean, Yao et al. (2017) generated 50% and 75% shade conditions by covering 2 m above ground level with black nets. They reported 4 and 2.5 fold decrease in photosynthetic photon flux density (PPFD) in 75%, and 50% shade compared to unshaded treatment, respectively. Liu et al. (2018) in a maize-soybean relay intercropping measured different light parameters when soybean seedlings were 16 d older, and informed a reduction of 82.7%, 50.0%, 65.5%, and 52.3% in RL, FRL, their ratios, and PPFD, respectively over normal light. Based on these values, we can say that our method of inducing low light conditions is comparable with previous studies in terms of effectiveness. It can replicate the same level of reduction in light composition due to defined net mesh size, and other controlled conditions, such as temperature and humidity. However, we can improve on consistency and validity of the experimental treatments throughout the study period by employing temperature and reliable light sensors capable of measuring not only light intensity but also related parameters (red/far-red components) alongwith installation of automated systems for irrigation and fertigation purposes.

### 4.2 Implication of ANOVA genotype-treatment interaction

The significant G×T interaction for the traits investigated (DTE, DTFFB, DTA, PH, IL, NPP, BPP, SDBPP, RDBPP, RSR and PTDB) indicated existence of different genotypic responses to altered light composition. These findings are in agreement with previous studies where significant genotype-by-treatment interaction had influenced PH (Getachew et al., 2015), DTA (Desai et al., 2016), SDBPP (Arif et al., 2021), RDBPP and PTDB (Nayak et al., 2010) in chickpea but under different conditions.

### 4.3 Effects of LL on photosynthetic response of parents

These were greater for PBA Slasher as indicated by 27.9% reduction in net photosynthetic rate compared to 12.8% of Sonali over NL treatment due to reduced PPFD under LL (Shrestha et al., 2019). LL severely affected this parental line in contrast to Sonali, and modified its true phenotypic expression through substantial increase in plant height (47.3% vs 27.8%), and internodes length (46.7% vs 19.0%), and decrease in branches (7.0% vs 0.0%), nodes (4.4% vs 2.4%), biomass of roots (83.3% vs 50.0%), shoots (50.0% vs 27.3%), root/shoot ratio (63.3% vs 21.7%), and total plant biomass (70.8% vs 36.8%), respectively. It also delayed seedling emergence (-17.6% vs 47.7%), and anthesis (- 2.4% vs 1.3%) in PBA Slasher as opposed to Sonali, wherein, these were induced much earlier in the season. The better response of Sonali to these conditions, especially for vegetative (PH, IL) and biomass (RDBPP, SDBPP, RSR, PTDB) indices, was attributed to superior light harvesting, net photosynthetic rate, and production of photosynthates (Cai, 2011; Shafiq et al., 2021). This is also evident from the performance of PBA Slasher under NL conditions, where it maintained 8.1% more Pn than Sonali, and excelled in crucial indices such as RDBPP, RSR, PTDB, and SDBPP by 33.3%, 29.9%, 20.8%, and 8.3%, and BPP and NPP by 57.0% and 10.1%, respectively. In maize and soybean, previous researchers also reported a significant reduction in photosynthetic capacity, and carbon gain of genotypes under suboptimal PPFD, and other light components (Pausch et al., 1991; Gao et al., 2017). Because, plants grown at different irradiance levels develop photosynthetic apparatus with altered features, so varied carbon fixation potential with overall reduced rate of photosynthesis (Bailey et al., 2001; Sun et al., 2014; Irving, 2015).

### 4.4 Effects of LL on plant phenology

Differences in days to emergence were observed among genotypes, which indicated varied thermal time requirement between two light treatments. The LL significantly promoted seed germination, resulting in, 3.7% less days to emerge from date of sowing than NL, suggesting minimum or no role of light, and had been reported previously in chickpea (Vignoli, 1936), and some other species (Chanyenga et al., 2012). Seed germination largely depends upon soil temperature, moisture, and seeding depth (Soltani et al., 2006). Since we kept all these requirements close to optimum in both the treatments, this accelerated emergence might be due to comparatively low temperature under shade which allowed seeds to imbibe enough water content to initiate germination earlier than NL (Tewfik, 2003; Ahmed et al., 2014).

The current study found that LL not only impeded first floral buds development but also commencement of anthesis, on average, by 3.1 and 8.4 more days, compared to NL, respectively. This is a consequence of drop in PPFD which substantially impacted photosynthetic process, hence, energy production and access to genotypes (Jiang and Egli, 1993; Cai, 2011). Previous studies on chickpea (Sandhu and Hodges, 1971; Samineni et al., 2020), and alfalfa (Lorenzo et al., 2019) also informed similar findings. Generally, plants employ two flowering strategies to counter shade. They either accelerate reproductive development as reported in *Oryza sative*, *Lotus japonicus*, and *Arabidopsis thaliana* (Cerdán and Chory, 2003; Ueoka-Nakanishi et al., 2011; Carriedo et al., 2016) or delay it, comparable to this, and previous other studies on sunflower, tomato, and alfalfa, possibly, as an adaptive strategy (Qin et al., 2022). The delayed flowering reported herein, allowed chickpea plants to intercept greater proportion of PAR, thus, more assimilates for sustaining vegetative and reproductive development, and production of biomass (Lake, 2017).

### 4.5 Effects of LL on shoot architecture

Suboptimal light causes reduction in thickness of leaf and palisade tissues, chlorophyll contents, and leaf area, resultantly, decreased light interception. This impacts the activity of gas exchange processes (stomatal density, conductance), consequently, inadequate CO_2_ transport. Further, transfer of electron from photosystem II to I is obstructed, and level of enzymes biosynthesis is modified. Moreover, reactive oxygen species (O_2_^-^, O_2_H, OH, & O) are produced, which interferes with the normal functioning of photosynthetic apparatus. These leads to reduction in rate of CO_2_ assimilation, net photosynthesis, and greater biomass partitioning to stems (Gong et al., 2015; Shafiq et al., 2021). We found that variation in RL : FRL ratio had enhanced plant height and internodal length of genotypes, on average, by 14.9 cm and 0.6 cm, respectively. Stem elongation is a well-known adaptive strategy in plants to altered light (Sessa et al., 2018; Wang et al., 2020). Increased plant height, and nodes length is a typical sign of shade avoidance syndrome (SAS), by which, plants elongate their stems in search of light. This resulted in weaker and slender stems, and had been reported by previous researchers in soybean (Green-Tracewicz et al., 2011; Zhang et al., 2011), sunflower, and Arabidopsis (Yang and Li, 2017). Low RL/FRL ratio, initially, promotes shade escape mechanism (Ballaré et al., 1990), and then inactivates phytochrome-interacting factors to produce increased level of auxins, which results in stem elongation (Li et al., 2012). Low PPFD is also responsible for this growth due to increased production level of gibberellin in hypocotyls, leaves, internodes, and shoots (Beall et al., 1996; Kurepin et al., 2007). Stem strength greatly depends upon synthesis of biochemical compounds, such as lignin, starch, pectin, sucrose, semi-fiber, and LL serves as a constraining factor in their production due to reduced enzymatic activities of phenylalanine, dehydrogenase, peroxidase, and ligase (Wu et al., 2017a; Hussain et al., 2019; Shafiq et al., 2021). Hormones, such as auxin and gibberellins, control LL induced plant growth and expansion (Yang and Li, 2017). This study has informed production of more NPP compared to NL conditions, similar to the report of Nico et al. (2015) in soybean. In contrast, Raai et al. (2020) in a study on winged beans found that non-shaded plants produced higher NPP than moderately shaded, and heavily shaded plants. For branches per plant, non-significant treatment effects were recorded. However, genotypic differences were recorded under both light treatments. Raai et al. (2020) in the same study also observed substantial variation in BPP, non-shaded being higher in BPP than shaded plants. This increase in NPP and BPP was due to delayed anthesis, and energy conserved over the extended time period, possibly, to endure challenging environments (Lorenzo et al., 2019).

### 4.6 Effects of LL on biomass production and partitioning

The ratio of biomass partitioning to above and below-ground plant parts is a way to study biomass allocation. Shoots represent the light-harvesting, energy-producing part, while roots are essential for nutrients, and water uptake from the soil. The larger the root system a plant develops, the higher its biomass and root-to-shoot ratio will be (Poorter and Nagel, 2000; Mašková and Herben, 2018). Among all the traits we investigated, LL effects were highest on biomass indices such as roots, shoots, root/shoot ratio, and plant total dry biomass. On average, it reduced RDBPP and SDBPP by 52.8% (0.4 g) and 28.2% (0.3 g), RSR by 36.3%, and PTDB by 38.4% (0.7 g) over NL treatment. Poor light intensity not only modified the true phenotypes through shifting of greater energy resources to vegetative parts (stem, nodes and branches) but also restricted root development. This resulted in insufficient carbon gain of roots, altering root/shoot ratio, and morphology of genotypes. These results are consistent with the previous studies on chickpea, which also revealed a reduction in these plant parts on reducing light artificially (Verghis et al., 1999; Li et al., 2010; Lake and Sadras, 2014). Similar observations were also reported in other crops like soybean, maize, and is a typical outcome of SAS when plants perceive low RL/FRL signal through phytochromes (Kurepin et al., 2007; Gao et al., 2017; Wu et al., 2017b; Wang et al., 2020). Biosynthesis of some of the phyto-hormones, such as auxin and ethylene increases under these conditions, which severely impacts root growth and development (Růžička et al., 2007). Underdeveloped roots could seriously affect tolerance of plants to water-deficit environments, and genotypes with compromised root system are more susceptible to drought, which is a severe issue in chickpea, particularly during reproductive phase (Pierik and Testerink, 2014; Blessing et al., 2018; Dreccer et al., 2018). Therefore, to mitigate these effects, genotypes as well as target traits are required to be identified for achieving sustainability in chickpea production.

### 4.7 Relationship among different plant traits

Across both light conditions, genotypes who took higher days to initiate flowering (DTA) produced greater RDBPP, SDBPP, RSR, and PTDB. These also exhibited slower growth rates as they took longer for DTE, and DTFFB development. Over the longer growing period, these genotypes attained higher PH with greater IL, NPP, and BPP. However, late flowering genotypes, as identified in NL and LL treatments (**Table 2**), performed better due to maximum light-harvesting, radiation use efficiency, and higher energy production (Li et al., 2008; Bai et al., 2016; Shafiq et al., 2021).

### 4.8 Genetic variation and heritability of plant traits captured

The existence of genetic diversity within or between crop species is indispensable for crop improvement against various stresses (Swarup et al., 2021). This offers plant breeders a chance to select for superior genotypes for use in breeding programs aimed at germplasm development, or release of cultivars for commercial cultivation (Naveed et al., 2020). The genotypic variation observed in this study was also impressive for some of the targeted traits, especially roots (0.10 to 1.01 g), shoots (0.22 to 1.44 g), total plant biomass (0.34 to 2.38 g), and days to anthesis (29.8 to 85.0 d) under LL conditions. For instance, RILs #107 (2.38 g), #4 (2.36 g), #107 (2.27 g), and #122 (2.01 g) outperformed parents, Sonali (1.23 g), PBA Slasher (0.74 g), and other lines (1.29 g) for total plant biomass as indicated by means. Likewise for root dry biomass per plant, RILs #93 (0.14 g), #151 (0.14 g), #143 (0.13 g), and #160 (0.12 g) failed to exceed parents, Sonali (0.42 g), PBA Slasher (0.20 g), and other genotypes (0.49 g). These results revealed presence of genetic diversity, continuous variation, and transgressive recombinants (+ve & -ve) for some of the parameters compared here of Sonali/PBA Slasher mapping population (Polania et al., 2017). Mapping populations are an excellent source of genetic diversity, and have been reported to possess transgressive segregants for salinity (Pushpavalli et al., 2015) and heat (Paul et al., 2018) tolerance in chickpea.

High broad-sense heritability estimates were recorded for indices such as DTE, DTFFB, DTA, PH, NPP, BPP, SDBPP, RDBPP, RSR and PTDB, under NL and LL conditions, except for IL. This suggested the least influence of environments on the expression of these parameters, and the potential of direct selection for further improvement under similar conditions (Hussain et al., 2016; Naveed et al., 2016).

### 4.9 Discrimination of genotypes for biomass traits

Among multivariate techniques, PCA biplot is the most effective method for assessing performance of genotypes, and interaction of traits. It has been extensively practiced to examine the association among traits in chickpea, and other field crops (Erdemci, 2018; Sharma et al., 2023). Biplots provided a new direction in understanding plant responses, and respective stress-tolerance mechanisms under various environmental conditions (Sivakumar et al., 2020; Rani et al., 2023).

In the present study, PCA biplot indicated strong associations among various plant traits under both the treatments, implying potential breeding strategies to emphasize for further improvement (Kushwah et al., 2022). The positive correlation of days to anthesis with total plant biomass (r= 0.88, 0.95), root biomass (r= 0.90, 0.93), and shoot biomass (r= 0.84, 0.94), as exhibited by biplot and Pearson’s correlation coefficients under NL and LL, respectively, suggested that genotypes with late maturity period produced plant parts, above and below ground, with greater biomass. Association of three biomass parameters, PTDB with RDBPP (r= 0.99, 0.99), SDBPP (r= 0.99, 0.98), and RDBPP with SDBPP (r= 0.95, 0.94), revealed correlated response with coefficients near to 1.0, and heritability values greater than 75.0%, indicating least environmental influence, and possibility of simultaneous improvement using different selection strategies (Rana et al., 2019). Direct selection, as early as, from F_2_ generation would be rewarding for progressing further in these traits (Sehrawat et al., 2012). Entries such as #147 (1_52), #161 (1_73), #153 (1_64), #86 (1_223), and #107 (1_245) revealed an increase of 43.0 to 25.3%, 50.7 to 28.3%, and 35.6 to 23.0% in total plant biomass, root biomass, and shoot biomass compared to respective trait means under NL conditions. Likewise, entries #107 (1_245), #77 (1_212), #97 (1_233), #122 (1_269), #4 (1_103), and #110 (1_248) surpassed trait means for biomass of roots, shoots, and total plant biomass by 47.9 to 38.0%, 44.4 to 34.4%, and 45.8 to 35.8%, respectively, under LL environment. Detailed analysis of these entries revealed that RILs viz., 1_52, 1_73, 1_64, 1_245, 1_103, 1_248, and 1_269, overall, produced more biomass (PTDB, RDBPP, SDBPP) with greater number of nodes, branches over 17.4 to 29.9% longer phenological period to start anthesis. Entry #107 (1_245) revealed phenotypic plasticity across both NL and LL with good scores for TPDB (3.12 vs 2.01 g), RDBPP (1.38 vs 0.79 g) and SDBPP (1.74 vs 1.22 g), therefore, could be used regardless of the specific environment (Sadras et al., 2016). This biplot also showed negative association of internodal length with all biomass traits, with heritability value of 36.1%, implying greater environmental effects, and delay in selection up to later generations, such as F_5_ or F_6_ through pedigree method would be rewarding (Khan et al., 2016).

All the promising RILs identified here could be utilized in different breeding schemes for creating new, and desirable recombinants, and developing shade-tolerant chickpea germplasm (Gommers et al., 2013; Naveed et al., 2015; Sulistyowati et al., 2016). Classical methods such as introduction, selection, and hybridization, are the most common breeding approaches used for selecting plant material with targeted features. However, these require greater time-period, and resources when traits of interest (such as root, shoot and total plant biomass etc.) are polygenic and correlated with each other. The selection process becomes even more complicated if there is a greater G×E interaction or trade-off (phenology and yield in chickpea etc.) among traits (Maqbool et al., 2017). To overcome these challenges, molecular techniques such as linkage maps and marker-assisted selection (MAS) could be used, being stable and unaffected by environmental fluctuations, and easily noticeable, regardless of growth stage. However, production of mapping populations is one of the basic requirement for constructing a linkage map and establishing marker-trait association (Collard et al., 2005). For this purpose, the RILs discriminated here could be used for developing segregating populations involving two or multiple parents. This could lead in identifying quantitative trait loci (QTL) associated with drought tolerance indices (root biomass, shoot biomass, and total plant biomass), and their incorporation through various MAS schemes. Completion of chickpea genome sequencing has further opened up avenues for crop improvement through omics techniques such as genomics, transcriptomics, and phenomics. We can combine QTL mapping with these methods to study the expression of genes, and molecular mechanisms regulating these parameters, and shade tolerance in genetic material so developed using these RILs. This would accelerate incorporation, and selection for shade tolerance traits (Mir et al., 2012; Dutta et al., 2018). Therefore, a multidisciplinary approach integrating genomics with breeding, coupled with precise phenotyping is suggested for conferring this type of stress in chickpea.

## 5 Conclusion

Modifications in optimum light conditions as revealed by reduced photosynthetic active radiation, red and far-red lights, and their ratios proved that our methodology of using mosquito net, had effectively, simulated low light conditions. The responses of chickpea genotypes to these changes were severe, as most of them altered their morphology with greater investment of available photosynthates on shoot growth at the expense of root development. Specifically, plants were slow in growth, produced greater plant heights, internodal lengths, and nodes per plant, however, with reduced root, shoot, and total plant biomass, and altered root to shoot ratios, possibly as an adaptive strategy, similar to the hypothesis of shade avoidance syndrome. Modifications in some biochemical and molecular processes might also be responsible for all these effects, but, were not part of our research, and would be of great worth in understanding shade effects, and possible mechanisms in future studies. Overall, low light effects were greater on biomass relevant parameters (root, shoot, their ratio, and total plant biomass), which are vital part of drought tolerance strategy in chickpea. Superior RILs identified through PC analysis, viz., 1_52, 1_73, 1_64, 1_245, 1_103, 1_248, and 1_269, produced highest TPDB with greater RDBPP, and SDBPP. These RILs, along with others identified in this study, could be the source material to develop light-insensitive chickpea cultivars through integrated breeding approaches.

## Data availability statement

The raw data supporting the conclusions of this article will be made available by the authors, without undue reservation.

## Author contributions

MN: Data curation, Investigation, Methodology, Writing – original draft. UB: Supervision, Writing – review & editing. BNK: Funding acquisition, Supervision, Writing – review & editing.
