# Supplementary material for: Impact of low light intensity on biomass partitioning and genetic diversity in a chickpea mapping population
Source: Front Plant Sci. 2024 Feb 1;15:1292753. doi: 10.3389/fpls.2024.1292753 (PMC10867217; doi:10.3389/fpls.2024.1292753)
Supplement: Supplementary file 1 [file DataSheet_1.docx]

Table S1 List of plant material comprising recombinant inbred lines (RILs) derived from a Sonali/PBA Slasher population, and commercially grown genotypes evaluated under two light treatments.

| **E** | **Name** | **GH** | **CP** | **PB** | **ST** | **E** | **Name** | **GH** | **CP** | **PB** | **ST** | **E** | **Name** | **GH** | **CP** | **PB** | **ST** | **E** | **Name** | **GH** | **CP** | **PB** | **ST** | **E** | **Name** | **GH** | **CP** | **PB** | **ST** |
| --- | --- | --- | --- | --- | --- | --- | --- | --- | --- | --- | --- | --- | --- | --- | --- | --- | --- | --- | --- | --- | --- | --- | --- | --- | --- | --- | --- | --- | --- |
| **#** |  |  |  |  |  | **#** |  |  |  |  |  | **#** |  |  |  |  |  | **#** |  |  |  |  |  | **#** |  |  |  |  |  |
| 1 | 1_100 | 3 | E | L | P | 37 | 1_165 | 2 | M | M | P | 73 | 1_209 | 2 | E | M | P | 109 | 1_247 | 2 | E | M | P | 145 | 1_50 | 3 | E | M | P |
| 2 | 1_101 | 3 | E | M | P | 38 | 1_166 | 2 | E | M | P | 74 | 1_21 | 2 | E | M | P | 110 | 1_248 | 3 | M | H | P | 146 | 1_51 | 3 | E | M | P |
| 3 | 1_102 | 2 | E | M | P | 39 | 1_167 | 2 | M | M | P | 75 | 1_210 | 3 | E | M | P | 111 | 1_249 | 2 | M | M | P | 147 | 1_52 | 3 | M | H | P |
| 4 | 1_103 | 2 | L | M | P | 40 | 1_168 | 2 | E | M | P | 76 | 1_211 | 3 | E | L | P | 112 | 1_25 | 3 | E | M | P | 148 | 1_55 | 3 | E | M | P |
| 5 | 1_105 | 2 | M | M | P | 41 | 1_169 | 1 | M | M | P | 77 | 1_212 | 1 | M | H | P | 113 | 1_251 | 2 | M | H | P | 149 | 1_59 | 3 | E | M | P |
| 6 | 1_108 | 3 | M | M | P | 42 | 1_17 | 2 | E | L | P | 78 | 1_213 | 3 | E | L | P | 114 | 1_252 | 1 | E | M | P | 150 | 1_6 | 3 | M | M | P |
| 7 | 1_110 | 2 | E | M | P | 43 | 1_170 | 3 | E | L | P | 79 | 1_214 | 2 | E | L | P | 115 | 1_257 | 3 | E | M | P | 151 | 1_60 | 3 | E | L | P |
| 8 | 1_115 | 3 | E | L | P | 44 | 1_171 | 2 | E | M | P | 80 | 1_215 | 1 | E | L | P | 116 | 1_258 | 3 | E | M | P | 152 | 1_61 | 3 | E | L | P |
| 9 | 1_116 | 2 | M | M | P | 45 | 1_172 | 2 | E | L | P | 81 | 1_217 | 2 | E | L | P | 117 | 1_259 | 3 | M | M | P | 153 | 1_64 | 3 | M | H | P |
| 10 | 1_119 | 2 | E | L | P | 46 | 1_176 | 2 | E | M | P | 82 | 1_218 | 2 | E | L | P | 118 | 1_26 | 3 | E | L | P | 154 | 1_66 | 2 | M | M | P |
| 11 | 1_120 | 2 | E | L | P | 47 | 1_178 | 2 | E | M | P | 83 | 1_22 | 2 | E | L | P | 119 | 1_260 | 3 | E | M | P | 155 | 1_67 | 2 | M | H | P |
| 12 | 1_121 | 2 | E | M | P | 48 | 1_179 | 3 | E | M | P | 84 | 1_221 | 2 | E | M | P | 120 | 1_263 | 3 | E | L | P | 156 | 1_68 | 2 | M | M | P |
| 13 | 1_124 | 3 | E | L | P | 49 | 1_18 | 3 | E | M | P | 85 | 1_222 | 2 | E | M | P | 121 | 1_265 | 2 | M | H | P | 157 | 1_69 | 3 | E | L | P |
| 14 | 1_132 | 2 | E | M | P | 50 | 1_180 | 2 | E | L | P | 86 | 1_223 | 2 | L | H | P | 122 | 1_269 | 1 | M | H | P | 158 | 1_7 | 3 | M | M | P |
| 15 | 1_133 | 3 | E | M | P | 51 | 1_181 | 3 | E | M | P | 87 | 1_224 | 4 | M | M | P | 123 | 1_27 | 2 | E | L | P | 159 | 1_71 | 3 | E | L | P |
| 16 | 1_136 | 3 | E | M | P | 52 | 1_182 | 3 | E | M | P | 88 | 1_225 | 1 | M | M | P | 124 | 1_270 | 2 | E | M | P | 160 | 1_72 | 3 | E | L | P |
| 17 | 1_138 | 3 | E | L | P | 53 | 1_184 | 3 | E | M | P | 89 | 1_226 | 2 | E | L | P | 125 | 1_271 | 2 | M | M | P | 161 | 1_73 | 1 | M | H | P |
| 18 | 1_139 | 2 | E | M | P | 54 | 1_19 | 3 | E | M | P | 90 | 1_227 | 2 | E | M | P | 126 | 1_273 | 3 | E | M | P | 162 | 1_76 | 4 | E | M | P |
| 19 | 1_14 | 3 | E | L | P | 55 | 1_190 | 2 | E | L | P | 91 | 1_228 | 2 | E | M | P | 127 | 1_28 | 3 | M | M | P | 163 | 1_77 | 3 | E | L | P |
| 20 | 1_140 | 2 | E | M | P | 56 | 1_191 | 3 | E | M | P | 92 | 1_229 | 2 | E | M | P | 128 | 1_29 | 3 | E | L | P | 164 | 1_79 | 3 | E | M | P |
| 21 | 1_144 | 3 | E | M | P | 57 | 1_192 | 2 | M | M | P | 93 | 1_23 | 3 | E | L | P | 129 | 1_3 | 3 | E | L | P | 165 | 1_8 | 3 | E | L | P |
| 22 | 1_145 | 2 | M | M | P | 58 | 1_193 | 3 | L | M | P | 94 | 1_230 | 2 | E | M | P | 130 | 1_32 | 3 | M | M | P | 166 | 1_81 | 3 | E | L | P |
| 23 | 1_146 | 2 | E | M | P | 59 | 1_194 | 1 | M | M | P | 95 | 1_231 | 1 | E | L | P | 131 | 1_33 | 3 | E | M | P | 167 | 1_83 | 3 | E | M | P |
| 24 | 1_147 | 3 | M | M | P | 60 | 1_195 | 2 | M | H | P | 96 | 1_232 | 3 | E | L | P | 132 | 1_34 | 2 | L | M | P | 168 | 1_84 | 2 | E | L | P |
| 25 | 1_148 | 2 | L | M | P | 61 | 1_197 | 2 | E | L | P | 97 | 1_233 | 1 | M | M | P | 133 | 1_35 | 2 | E | L | P | 169 | 1_86 | 4 | E | M | P |
| 26 | 1_15 | 3 | E | M | P | 62 | 1_198 | 2 | E | M | P | 98 | 1_234 | 3 | E | L | P | 134 | 1_36 | 2 | E | M | P | 170 | 1_87 | 2 | E | L | P |
| 27 | 1_150 | 1 | E | M | P | 63 | 1_199 | 2 | E | M | P | 99 | 1_235 | 1 | M | M | P | 135 | 1_37 | 3 | E | L | P | 171 | 1_89 | 2 | E | M | P |
| 28 | 1_154 | 2 | E | M | P | 64 | 1_2 | 3 | E | M | P | 100 | 1_236 | 3 | E | L | P | 136 | 1_38 | 2 | M | M | P | 172 | 1_90 | 3 | E | M | P |
| 29 | 1_155 | 2 | E | L | P | 65 | 1_200 | 1 | E | M | P | 101 | 1_24 | 3 | L | M | P | 137 | 1_39 | 2 | E | M | P | 173 | 1_96 | 2 | E | M | P |
| 30 | 1_156 | 2 | E | L | P | 66 | 1_201 | 2 | M | M | P | 102 | 1_240 | 2 | E | M | P | 138 | 1_4 | 3 | E | M | P | 174 | 1_97 | 3 | L | H | P |
| 31 | 1_157 | 2 | E | M | P | 67 | 1_202 | 3 | E | M | P | 103 | 1_241 | 3 | M | M | P | 139 | 1_44 | 3 | E | L | P | 175 | 1_99 | 1 | E | M | P |
| 32 | 1_159 | 1 | E | M | P | 68 | 1_203 | 3 | E | M | P | 104 | 1_242 | 2 | M | M | P | 140 | 1_46 | 2 | M | M | P | 176 | 25001 | 2 | M | M | PL |
| 33 | 1_160 | 2 | E | M | P | 69 | 1_204 | 2 | M | M | P | 105 | 1_243 | 3 | E | M | P | 141 | 1_47 | 3 | E | M | P | 177 | Sonali | 2 | M | M | PL/CG |
| 34 | 1_161 | 2 | E | M | P | 70 | 1_205 | 2 | L | H | P | 106 | 1_244 | 2 | E | L | P | 142 | 1_48 | 2 | M | M | P | 178 | PBA Slasher | 3 | E | M | PL/CG |
| 35 | 1_162 | 2 | E | L | P | 71 | 1_206 | 2 | E | M | P | 107 | 1_245 | 2 | L | H | P | 143 | 1_49 | 2 | E | L | P | 179 | PBA Seamer | 3 | M | M | CG |
| 36 | 1_164 | 2 | E | M | P | 72 | 1_208 | 1 | M | M | P | 108 | 1_246 | 2 | M | H | P | 144 | 1_5 | 3 | M | M | P | 180 | PBA Striker | 3 | E | M | CG |

E, Entry; GH, Growth habit; 1 = Erect (0–15°), 2 = Semi-erect (15–25°), 3 = Semi-spreading (25–60°), 4 = Spreading (60–80°), 5 = Prostrate (branches sidling above the ground level); ST, Status; PL, Parental line; P, Progeny; CG, Commercial genotype; CP, Cropping period; E = Early maturing; M = Medium maturity; L = Late maturing; PB, Plant biomass; L = Low biomass; M = Medium biomass; H = High biomass

Table S2 List of plant traits and the procedure followed to measure under natural light (NL) and low light (LL) treatments.

| **Trait** | **Methodology** |
| --- | --- |
| Days to emergence (DTE) | Days taken from sowing of seeds to the date when the plumule broke through the soil surface (cracking stage) were referred to as days to emergence. |
| Days to first floral bud (DTFFB) | The days taken from sowing of seeds to the appearance of the first floral bud on the shoot apex of individual plants were called days to first floral bud. |
| Days to anthesis (DTA) | From the sowing of seeds to the emergence of three fully developed flowers, the days taken by individual plants were determined as days to anthesis. |
| Plant height (PH) | Height of individual plants from the base of the main stem to apical meristem on the day it reached anthesis was referred to as plant height at anthesis. |
| Nodes per plant (NPP) | Node numbers on the main stem of each of the individual plants once they reached the anthesis stage were recorded as nodes per plant. |
| Internodal length (IL) | It was estimated by dividing plant height recorded at anthesis by the number of nodes of the plant. |
| Branches per plant (BPP) | The number of branches developed on the main stem of an individual plant at the anthesis stage was referred to as branches per plant. |
| Shoot dry biomass per plant (SDBPP) | The shoots (above-ground plant part) of individual plants were separated from roots by cutting plants at the base of the stem once they reached the anthesis stage. The fresh shoots were put in a separate paper bag, and then dried in a dehydrator at 70° C for 72 hours before weighing (g). |
| Root dry biomass per plant (RDBPP) | For loosening soil from the roots, the individual pots were first soaked for 1 hour in a bucket filled with water. Afterwards, roots were washed to remove the soil, put into separate bags, and dried at 70° C for at least 72 hours in a dehydrator. The dried roots were later weighed (g) using an electric balance. |
| Root/shoot ratio (RSR) | The root to shoot ratio (RSR) was obtained by dividing the individual root dry biomass with the shoot dry biomass of the respective plant and the fraction multiplied by 100. |
| Plant total dry biomass (PTDB) | The root and shoot biomass obtained of individual plants were summed-up to determine total plant dry biomass. |

Table S3 Composition of available light to plants at five different stages (S) of experiment inside a glasshouse under natural light (NL), and low light (LL) treatments (T).

| **T** | **S** | **PAR**  μmol m^-2^ s^-1^ | **RL**  μmol m^-2^ s^-1^ | **FRL**  μmol m^-2^ s^-1^ | **RL/FRL** |
| --- | --- | --- | --- | --- | --- |
| NL | BS | 1085.0 | 74.1 | 58.6 | 1.28 |
| LL |  | 339.0 | 23.6 | 18.9 | 1.24 |
| NL | AS | 713.3 | 94.1 | 80.8 | 1.17 |
| LL |  | 196.5 | 27.5 | 23.9 | 1.15 |
| NL | 3-LS | 741.2 | 95.7 | 84.4 | 1.15 |
| LL |  | 191.2 | 26.2 | 24.1 | 1.10 |
| NL | 4-LS | 757.3 | 98.9 | 85.8 | 1.17 |
| LL |  | 212.4 | 30.5 | 26.0 | 1.17 |
| NL | AS | 841.8 | 108.0 | 96.3 | 1.14 |
| LL |  | 206.2 | 28.9 | 25.8 | 1.12 |
| *T Means* |  |  |  |  |  |
| NL |  | 827.7 | 94.2 | 81.2 | 1.18 |
| LL |  | 229.1 | 27.3 | 23.7 | 1.16 |
| *% decrease* |  | *72%* | *71%* | *71%* | *2%* |
| *P* value |  | < 0.001 | < 0.001 | < 0.001 | 0.025 |
| SED |  | 31.2 | 3.6 | 3.4 | 0.01 |
| LSD (0.05) |  | 61.4 | 7.2 | 6.7 | 0.02 |

BS, Before sowing; AS, After sowing; 3-LS, 3-Leaf stage; 4-LS, 4-Leaf stage; AS, Anthesis stage; SED, Standard error of difference; LSD, Least significant differences.

Table S4 Impact of natural light (NL), and low light (LL) treatments (T) on photosynthetic rate (Pn) of parents recorded at three different growth stages (GS) inside a glasshouse.

| **Fixed term** | **T** | **GS** | **G** | **T×GS** | **G×T** | **G×GS** | **T×GS×G** |
| --- | --- | --- | --- | --- | --- | --- | --- |
| Pn  (μmol m^-2^ s^-1^) | 38.1 | 0.01 | 0.00 | 0.00 | 6.37 | 0.00 | 0.00 |
|  | *** |  |  |  | * |  |  |
| G×T means |  |  |  |  |  |  |  |
| Sonali | NL | 3-LS |  |  | 24.9 |  |  |
|  |  | 4-LS |  |  | 25.0 |  |  |
|  |  | AS |  |  | 25.2 |  |  |
|  | LL | 3-LS |  |  | 21.7 |  |  |
|  |  | 4-LS |  |  | 21.8 |  |  |
|  |  | AS |  |  | 21.9 |  |  |
| PBA Slasher | NL | 3-LS |  |  | 27.1 |  |  |
|  |  | 4-LS |  |  | 27.2 |  |  |
|  |  | AS |  |  | 27.4 |  |  |
|  | LL | 3-LS |  |  | 19.4 |  |  |
|  |  | 4-LS |  |  | 19.5 |  |  |
|  |  | AS |  |  | 19.7 |  |  |
| *T means* |  |  |  |  |  |  |  |
| Sonali | NL |  |  |  | 25.0bc |  |  |
|  | LL |  |  |  | 21.8ab |  |  |
| PBA Slasher | NL |  |  |  | 27.2c |  |  |
|  | LL |  |  |  | 19.6a |  |  |
| SED |  |  |  |  | 1.25 |  |  |
| LSD (0.05) |  |  |  |  | 2.46 |  |  |

G, Genotypes; BS, Before sowing; AS, After sowing; 3-LS, 3-Leaf stage; 4-LS, 4-Leaf stage; AS, Anthesis stage. *** & * represent significant differences at *P* < 0.001 and *P* < 0.05, respectively. Values without an asterisk (*) mark indicate non-significance at *P* > 0.05. Different superscript letters for T means represent significant (*P* < 0.05) differences as per Tukey's posthoc test.

Table S5 Variance analysis of different plant traits recorded for 180 genotypes (G) inside a glasshouse under natural light (NL), and low light (LL) treatments (T).

| **Traits** | **G** | **T** | **G×T interaction** |
| --- | --- | --- | --- |
| DTE | 4926.6*** | 115.3*** | 1569.8*** |
| DTFFB | 93470.9*** | 4079.4*** | 17737.9*** |
| DTA | 118329.9*** | 10057.8*** | 21817.4*** |
| PH | 16689.9*** | 6192.4*** | 3282.9*** |
| IL | 1232.3*** | 9828.9*** | 594.4*** |
| NPP | 8517.6*** | 91.2*** | 1526.6*** |
| BPP | 18651.3*** | 0.7 | 4746.8*** |
| SDBPP | 101706.0*** | 78575.0*** | 24415.5*** |
| RDBPP | 173644.5*** | 132276.1*** | 42809.4*** |
| RSR | 29285.9*** | 32538.8*** | 9026.6*** |
| PTDB | 259025.8*** | 241679.1*** | 59694.5*** |

*** represents significant differences at *P* < 0.001. Values without an asterisk (*) mark indicate non-significance at *P* > 0.05. DTE, Days to emergence (days); DTFFB, Days to first floral bud (days); DTA, Days to anthesis (days); PH, Plant height (cm); IL, Internodal length (cm); NPP, Nodes per plant; BPP, Branches per plant; SDBPP, Shoot dry biomass per plant (g); RDBPP, Root dry biomass per plant (g); RSR, Root/shoot ratio (%); PTDB, Plant total dry biomass (g).

Table S6 Pearson’s correlation coefficients among various plant traits recorded in Sonali/PBA Slasher RILs population under natural light (NL) and low light (LL) treatments inside a glasshouse.

| **Traits** | **DTE** | **DTFFB** | **DTA** | **PH** | **IL** | **NPP** | **BPP** | **SDBPP** | **RDBPP** | **RSR** |
| --- | --- | --- | --- | --- | --- | --- | --- | --- | --- | --- |
| NL |  |  |  |  |  |  |  |  |  |  |
| DTFFB | 0.63*** |  |  |  |  |  |  |  |  |  |
| DTA | 0.76*** | 0.90*** |  |  |  |  |  |  |  |  |
| PH | 0.65*** | 0.92*** | 0.94*** |  |  |  |  |  |  |  |
| IL | 0.25*** | 0.15 | 0.17* | 0.27*** |  |  |  |  |  |  |
| NPP | 0.58*** | 0.91*** | 0.93*** | 0.95*** | -0.04 |  |  |  |  |  |
| BPP | 0.44*** | 0.62*** | 0.60*** | 0.65*** | 0.21*** | 0.58*** |  |  |  |  |
| SDBPP | 0.60*** | 0.88*** | 0.84*** | 0.92*** | 0.19* | 0.89*** | 0.66*** |  |  |  |
| RDBPP | 0.60*** | 0.91*** | 0.90*** | 0.95*** | 0.11 | 0.96*** | 0.63*** | 0.95*** |  |  |
| RSR | 0.48*** | 0.78*** | 0.81*** | 0.80*** | -0.10 | 0.89*** | 0.41*** | 0.70*** | 0.88*** |  |
| PTDB | 0.61*** | 0.91*** | 0.88*** | 0.95*** | 0.15* | 0.94*** | 0.65*** | 0.99*** | 0.99*** | 0.80*** |
| LL |  |  |  |  |  |  |  |  |  |  |
| DTFFB | 0.85*** |  |  |  |  |  |  |  |  |  |
| DTA | 0.80*** | 0.91*** |  |  |  |  |  |  |  |  |
| PH | 0.77*** | 0.91*** | 0.97*** |  |  |  |  |  |  |  |
| IL | 0.22** | 0.24** | 0.18* | 0.22** |  |  |  |  |  |  |
| NPP | 0.68*** | 0.82*** | 0.94*** | 0.95*** | -0.08 |  |  |  |  |  |
| BPP | 0.84*** | 0.95*** | 0.94*** | 0.94*** | 0.25*** | 0.87*** |  |  |  |  |
| SDBPP | 0.80*** | 0.88*** | 0.94*** | 0.96*** | 0.18* | 0.92*** | 0.94*** |  |  |  |
| RDBPP | 0.84*** | 0.94*** | 0.93*** | 0.92*** | 0.24*** | 0.84*** | 0.96*** | 0.94*** |  |  |
| RSR | 0.76*** | 0.91*** | 0.90*** | 0.86*** | 0.20** | 0.80*** | 0.90*** | 0.84*** | 0.96*** |  |
| PTDB | 0.83*** | 0.93*** | 0.95*** | 0.95*** | 0.22** | 0.89*** | 0.97*** | 0.98*** | 0.99*** | 0.92*** |

*** represents significance at *P* < 0.001. Values without an asterisk (*) mark indicate non-significance at *P* > 0.05. DTE, Days to emergence (days); DTFFB, Days to first floral bud (days); DTA, Days to anthesis (days); PH, Plant height (cm); IL, Internodal length (cm); NPP, Nodes per plant; BPP, Branches per plant; SDBPP, Shoot dry biomass per plant (g); RDBPP, Root dry biomass per plant (g); RSR, Root/shoot ratio (%); PTDB, Plant total dry biomass (g).

Table S7 Principal Component analysis of various plant traits of Sonali/PBA Slasher RILs population including commercial genotypes grown in a glasshouse under natural light (NL) and low light (LL) treatments.

| **Variables** | **PC1** | **PC2** |
| --- | --- | --- |
| Eigenvalue | 6.47 | 3.14 |
| Variability (%) | 58.9 | 28.6 |
| Cumulative % | 58.9 | 87.4 |
| Traits | PC1 | PC2 |
| DTE | 30.5 | 5.50 |
| DTFFB | 34.3 | 21.9 |
| DTA | 31.8 | 30.6 |
| PH | 20.3 | 47.3 |
| IL | -3.15 | 52.0 |
| NPP | 34.5 | 17.1 |
| BPP | 32.3 | 11.4 |
| SDBPP | 33.5 | -24.9 |
| RDBPP | 32.9 | -29.2 |
| RSR | 29.8 | -31.4 |
| PTDB | 33.7 | -27.2 |

DTE, Days to emergence (days); DTFFB, Days to first floral bud (days); DTA, Days to anthesis (days); PH, Plant height (cm); IL, Internodal length (cm); NPP, Nodes per plant; BPP, Branches per plant; SDBPP, Shoot dry biomass per plant (g); RDBPP, Root dry biomass per plant (g); RSR, Root/shoot ratio (%); PTDB, Plant total dry biomass (g).
